# 2-(4-Fluoro­phen­yl)-5-iodo-3-isopropyl­sulfonyl-1-benzofuran

**DOI:** 10.1107/S1600536812043218

**Published:** 2012-10-20

**Authors:** Hong Dae Choi, Pil Ja Seo, Uk Lee

**Affiliations:** a Department of Chemistry, Dongeui University, San 24 Kaya-dong, Busanjin-gu, Busan 614-714, Republic of Korea; b Department of Chemistry, Pukyong National University, 599-1 Daeyeon 3-dong, Nam-gu, Busan 608-737, Republic of Korea

## Abstract

There are two symmetry-independent mol­ecules, *A* and *B*, in the asymmetric unit of the title compound, C_17_H_14_FIO_3_S. The dihedral angle formed by the 4-fluoro­phenyl ring and the mean plane [r.m.s. deviation = 0.013 (2) Å in mol­ecule *A* and 0.016 (2) Å in mol­ecule *B*] of the benzofuran fragment is 57.71 (7)° in mol­ecule *A* and 44.95 (7)° in mol­ecule *B*. In the crystal, mol­ecules are linked by weak C—H⋯O hydrogen bonds and I⋯O contacts [I⋯O = 3.3646 (15) and 3.2354 (14) Å], forming a three--dimensional network.

## Related literature

For background information and the crystal structures of related compounds, see: Choi *et al.* (2010*a* ▶,*b* ▶). For a review of halogen bonding, see: Politzer *et al.* (2007 ▶).
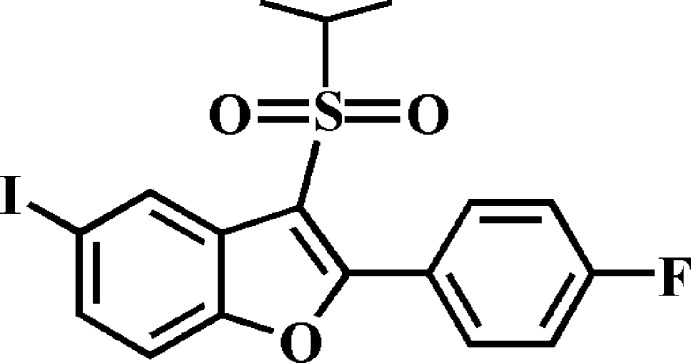


## Experimental

### 

#### Crystal data

C_17_H_14_FIO_3_S
*M* *_r_* = 444.24
Triclinic, 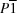

*a* = 11.7419 (2) Å
*b* = 12.8226 (2) Å
*c* = 12.8474 (2) Å
α = 66.576 (1)°
β = 82.703 (1)°
γ = 67.769 (1)°
*V* = 1642.36 (5) Å^3^
*Z* = 4
Mo *K*α radiation
μ = 2.10 mm^−1^
*T* = 173 K
0.31 × 0.22 × 0.22 mm

#### Data collection

Bruker SMART APEXII CCD diffractometer
Absorption correction: multi-scan (*SADABS*; Bruker, 2009 ▶) *T*_min_ = 0.594, *T*_max_ = 0.746
30779 measured reflections
8149 independent reflections
7224 reflections with *I* > 2σ(*I*)
*R*_int_ = 0.031

#### Refinement

*R*[*F* ^2^ > 2σ(*F* ^2^)] = 0.025
*wR*(*F* ^2^) = 0.060
*S* = 1.01
8149 reflections
419 parameters
H-atom parameters constrained
Δρ_max_ = 0.65 e Å^−3^
Δρ_min_ = −0.88 e Å^−3^

### 

Data collection: *APEX2* (Bruker, 2009 ▶); cell refinement: *SAINT* (Bruker, 2009 ▶); data reduction: *SAINT*; program(s) used to solve structure: *SHELXS97* (Sheldrick, 2008 ▶); program(s) used to refine structure: *SHELXL97* (Sheldrick, 2008 ▶); molecular graphics: *ORTEP-3* (Farrugia, 1997 ▶) and *DIAMOND* (Brandenburg, 1998 ▶); software used to prepare material for publication: *SHELXL97*.

## Supplementary Material

Crystal structure: contains datablock(s) global, I. DOI: 10.1107/S1600536812043218/ld2079sup1.cif

Structure factors: contains datablock(s) I. DOI: 10.1107/S1600536812043218/ld2079Isup2.hkl

Supplementary material file. DOI: 10.1107/S1600536812043218/ld2079Isup3.cml

Additional supplementary materials:  crystallographic information; 3D view; checkCIF report

## Figures and Tables

**Table 1:** Hydrogen-bond geometry (Å, °)

*D*—H⋯*A*	*D*—H	H⋯*A*	*D*⋯*A*	*D*—H⋯*A*
C6—H6⋯O1^i^	0.95	2.56	3.418 (2)	150
C15—H15⋯O2^ii^	1.00	2.52	3.520 (3)	177
C30—H30⋯O2^iii^	0.95	2.58	3.463 (3)	155
C31—H31⋯O3^iii^	0.95	2.58	3.260 (2)	129

Symmetry codes: (i) 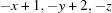; (ii) 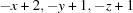; (iii) 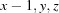.

**Fig. 1.**
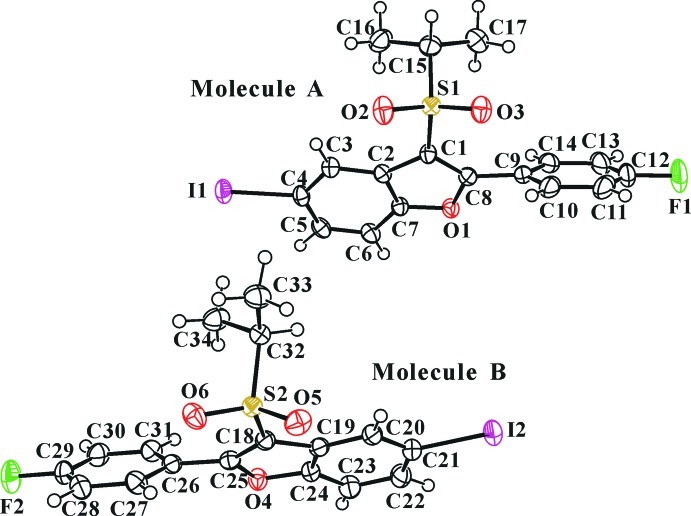
The molecular structure of the title compound with the atom numbering scheme. Displacement ellipsoids are drawn at the 50% probability level. H atoms are presented as small spheres of arbitrary radius.

**Fig. 2.**
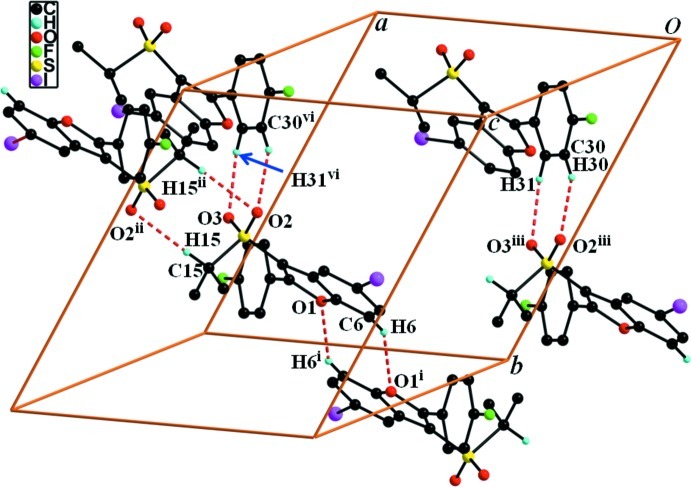
A view of the C—H···O interactions (dotted lines) in the crystal structure of the title compound. H atoms non-participating in hydrogen-bonding were omitted for clarity. [Symmetry codes: (i) - x + 1, - y + 2, - z; (ii) - x + 2, - y + 1, - z + 1; (iii) x - 1, y, z; (vi) x + 1, y, z.]

**Fig. 3.**
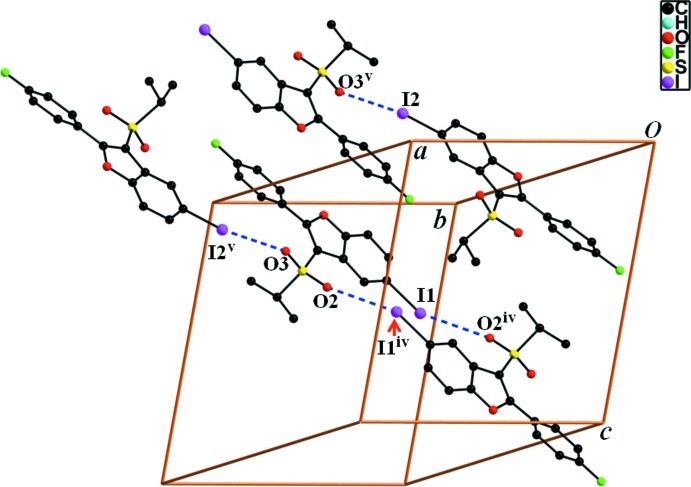
A view of the I···O interactions (dotted lines) in the crystal structure of the title compound. All H atoms were omitted for clarity. [Symmetry codes: (iv) - x + 1, - y + 1, - z + 1; (v) - x + 2, - y + 1, - z.]

## supplementary crystallographic information

### Comment

As a part of our ongoing study of 2-(4-fluorophenyl)-5-iodo-1-benzofuran derivatives containing ethylsulfinyl (Choi *et al.*, 2010*a*) and isopropylsulfinyl (Choi *et al.*, 2010*b*) substituents in 3-position, we report herein the crystal structure of the title compound which crystallizes with two symmetrically independent molecules, A and B, in the asymmetric unit.

In the title molecule (Fig. 1), the benzofuran unit is essentially planar, with a mean deviation of 0.013 (2)and 0.016 (2) Å, for A and B, respectively, from the least-squares plane defined by its nine constituent atoms. The dihedral angles between the 4-fluorophenyl ring and the mean plane of the benzofuran fragment are 57.71 (7)° in molecule A and 44.95 (7)° in molecule B, respectively. In the crystal packing (Fig. 2), molecules are linked by weak C—H···O hydrogen bonds (Table 1). The crystal packing (Fig. 3) also features I···O halogen-bondings between the iodine and the oxygen of the O═S═O unit [I1···O2^iv^ = 3.3646 (15) Å , C4—I1···O2^iv^ = 152.77 (6)° & I2···O3^v^ = 3.2354 (14) Å, C21—I2···O3^v^ = 170.66 (6)°] (Politzer *et al.*, 2007).

### Experimental

3-Chloroperoxybenzoic acid (77%, 381 mg, 1.7 mmol) was added in small portions to a stirred solution of 2-(4-fluorophenyl)-5-iodo-3-isopropylsulfanyl-1-benzofuran (330 mg, 0.8 mmol) in dichloromethane (50 mL) at 273 K. After being stirred at room temperature for 10h, the mixture was washed with saturated sodium bicarbonate solution, and the organic layer was separated, dried over magnesium sulfate, filtered and concentrated at reduced pressure. The residue was purified by column chromatography (benzene) to afford the title compound as a colorless solid [yield 72%, m.p. 422–423 K; *R*f = 0.65 (benzene)]. Single crystals suitable for X-ray diffraction were prepared by slow evaporation of a solution of the title compound in acetone at room temperature.

### Refinement

All H atoms were geometrically positioned and refined using a riding model, with C—H = 0.95 Å for aryl, 1.00 Å for methine and 0.98 Å for methyl H atoms, respectively. *U*_iso_(H) = 1.2*U*_eq_(C) for aryl, methine, and 1.5*U*_eq_(C) for methyl H atoms. The positions of methyl hydrogens were optimized rotationally.

### Crystal data

**Table:**

C_17_H_14_FIO_3_S	*Z* = 4
*M**_r_* = 444.24	*F*(000) = 872
Triclinic, *P*1	*D*_x_ = 1.797 Mg m^−^^3^
Hall symbol: -P 1	Melting point: 422.5 K
*a* = 11.7419 (2) Å	Mo *K*α radiation, λ = 0.71073 Å
*b* = 12.8226 (2) Å	Cell parameters from 9874 reflections
*c* = 12.8474 (2) Å	θ = 2.5–28.3°
α = 66.576 (1)°	µ = 2.10 mm^−^^1^
β = 82.703 (1)°	*T* = 173 K
γ = 67.769 (1)°	Block, colourless
*V* = 1642.36 (5) Å^3^	0.31 × 0.22 × 0.22 mm

### Data collection

**Table:**

Bruker SMART APEXII CCD diffractometer	8149 independent reflections
Radiation source: rotating anode	7224 reflections with *I* > 2σ(*I*)
Graphite multilayer monochromator	*R*_int_ = 0.031
Detector resolution: 10.0 pixels mm^-1^	θ_max_ = 28.3°, θ_min_ = 1.7°
φ and ω scans	*h* = −15→15
Absorption correction: multi-scan (*SADABS*; Bruker, 2009)	*k* = −17→16
*T*_min_ = 0.594, *T*_max_ = 0.746	*l* = −17→17
30779 measured reflections	

### Refinement

**Table:**

Refinement on *F*^2^	Primary atom site location: structure-invariant direct methods
Least-squares matrix: full	Secondary atom site location: difference Fourier map
*R*[*F*^2^ > 2σ(*F*^2^)] = 0.025	Hydrogen site location: difference Fourier map
*wR*(*F*^2^) = 0.060	H-atom parameters constrained
*S* = 1.01	*w* = 1/[σ^2^(*F*_o_^2^) + (0.0258*P*)^2^ + 0.967*P*] where *P* = (*F*_o_^2^ + 2*F*_c_^2^)/3
8149 reflections	(Δ/σ)_max_ = 0.001
419 parameters	Δρ_max_ = 0.65 e Å^−^^3^
0 restraints	Δρ_min_ = −0.88 e Å^−^^3^

### Special details

**Table:**

Geometry. All esds (except the esd in the dihedral angle between two l.s. planes) are estimated using the full covariance matrix. The cell esds are taken into account individually in the estimation of esds in distances, angles and torsion angles; correlations between esds in cell parameters are only used when they are defined by crystal symmetry. An approximate (isotropic) treatment of cell esds is used for estimating esds involving l.s. planes.
Refinement. Refinement of F^2^ against ALL reflections. The weighted R-factor wR and goodness of fit S are based on F^2^, conventional R-factors R are based on F, with F set to zero for negative F^2^. The threshold expression of F^2^ > 2sigma(F^2^) is used only for calculating R-factors(gt) etc. and is not relevant to the choice of reflections for refinement. R-factors based on F^2^ are statistically about twice as large as those based on F, and R- factors based on ALL data will be even larger.

### Fractional atomic coordinates and isotropic or equivalent isotropic displacement parameters (Å^2^)

**Table:**

	*x*	*y*	*z*	*U*_iso_*/*U*_eq_	
I1	0.326089 (13)	0.662386 (14)	0.466164 (12)	0.03553 (5)	
I2	0.737096 (13)	0.416299 (14)	−0.189163 (12)	0.03660 (5)	
S1	0.88239 (4)	0.60922 (4)	0.32898 (4)	0.02482 (10)	
S2	0.54126 (4)	0.09203 (4)	0.24522 (4)	0.02417 (10)	
F1	1.12323 (18)	0.8849 (3)	−0.18380 (15)	0.0963 (8)	
F2	−0.06003 (14)	0.13892 (15)	0.42793 (13)	0.0525 (4)	
O1	0.64958 (12)	0.85328 (12)	0.07633 (11)	0.0260 (3)	
O2	0.84377 (14)	0.51275 (13)	0.40782 (13)	0.0352 (3)	
O3	0.99193 (13)	0.57644 (13)	0.26654 (13)	0.0328 (3)	
O4	0.26036 (12)	0.35894 (13)	0.04663 (12)	0.0274 (3)	
O5	0.64518 (14)	0.04309 (14)	0.18388 (13)	0.0350 (3)	
O6	0.48419 (14)	0.01133 (14)	0.32324 (13)	0.0342 (3)	
C1	0.75915 (17)	0.70959 (17)	0.23334 (16)	0.0230 (4)	
C2	0.62962 (17)	0.73192 (16)	0.25781 (16)	0.0221 (4)	
C3	0.56186 (18)	0.68568 (17)	0.35032 (17)	0.0249 (4)	
H3	0.6008	0.6255	0.4205	0.030*	
C4	0.43510 (18)	0.73193 (17)	0.33472 (17)	0.0253 (4)	
C5	0.37556 (18)	0.82104 (18)	0.23277 (18)	0.0280 (4)	
H5	0.2884	0.8496	0.2260	0.034*	
C6	0.44196 (18)	0.86794 (18)	0.14196 (17)	0.0276 (4)	
H6	0.4030	0.9292	0.0722	0.033*	
C7	0.56748 (17)	0.82128 (17)	0.15782 (16)	0.0236 (4)	
C8	0.76507 (18)	0.78429 (18)	0.12401 (16)	0.0243 (4)	
C9	0.86352 (19)	0.8079 (2)	0.04658 (16)	0.0293 (4)	
C10	0.9528 (2)	0.7157 (3)	0.01943 (19)	0.0390 (5)	
H10	0.9541	0.6346	0.0539	0.047*	
C11	1.0412 (2)	0.7437 (3)	−0.0595 (2)	0.0563 (8)	
H11	1.1036	0.6819	−0.0792	0.068*	
C12	1.0364 (3)	0.8610 (4)	−0.1074 (2)	0.0602 (9)	
C13	0.9495 (3)	0.9538 (3)	−0.0828 (2)	0.0540 (8)	
H13	0.9490	1.0346	−0.1184	0.065*	
C14	0.8622 (2)	0.9275 (2)	−0.00479 (18)	0.0396 (5)	
H14	0.8009	0.9905	0.0142	0.048*	
C15	0.90404 (19)	0.6917 (2)	0.40407 (17)	0.0312 (4)	
H15	0.9739	0.6348	0.4598	0.037*	
C16	0.7904 (2)	0.7301 (2)	0.47178 (19)	0.0403 (5)	
H16A	0.8057	0.7705	0.5163	0.060*	
H16B	0.7725	0.6579	0.5229	0.060*	
H16C	0.7199	0.7865	0.4196	0.060*	
C17	0.9410 (2)	0.7973 (2)	0.3244 (2)	0.0382 (5)	
H17A	0.8714	0.8591	0.2732	0.057*	
H17B	1.0108	0.7679	0.2798	0.057*	
H17C	0.9648	0.8333	0.3687	0.057*	
C18	0.43388 (17)	0.21427 (17)	0.14277 (16)	0.0240 (4)	
C19	0.46896 (18)	0.29646 (17)	0.04048 (16)	0.0242 (4)	
C20	0.57969 (18)	0.30585 (19)	−0.00800 (17)	0.0267 (4)	
H20	0.6566	0.2501	0.0289	0.032*	
C21	0.57303 (19)	0.3995 (2)	−0.11194 (17)	0.0292 (4)	
C22	0.4612 (2)	0.4823 (2)	−0.16880 (19)	0.0336 (5)	
H22	0.4609	0.5442	−0.2410	0.040*	
C23	0.3503 (2)	0.4745 (2)	−0.12006 (18)	0.0324 (4)	
H23	0.2732	0.5295	−0.1570	0.039*	
C26	0.21380 (17)	0.22260 (18)	0.22001 (16)	0.0249 (4)	
C24	0.35885 (18)	0.38246 (19)	−0.01535 (17)	0.0266 (4)	
C25	0.30780 (18)	0.25643 (17)	0.14309 (16)	0.0245 (4)	
C27	0.21988 (19)	0.10232 (19)	0.27135 (18)	0.0292 (4)	
H27	0.2879	0.0395	0.2582	0.035*	
C28	0.1273 (2)	0.0737 (2)	0.34156 (18)	0.0327 (4)	
H28	0.1309	−0.0082	0.3767	0.039*	
C29	0.0304 (2)	0.1664 (2)	0.35910 (18)	0.0334 (5)	
C30	0.0208 (2)	0.2862 (2)	0.31029 (19)	0.0343 (5)	
H30	−0.0474	0.3481	0.3246	0.041*	
C31	0.11328 (19)	0.31421 (19)	0.23962 (18)	0.0295 (4)	
H31	0.1082	0.3965	0.2042	0.035*	
C32	0.58508 (18)	0.16381 (18)	0.31956 (17)	0.0265 (4)	
H32	0.6135	0.2289	0.2633	0.032*	
C33	0.6918 (2)	0.0688 (2)	0.40173 (19)	0.0364 (5)	
H33A	0.7214	0.1084	0.4381	0.055*	
H33B	0.7588	0.0301	0.3601	0.055*	
H33C	0.6638	0.0069	0.4598	0.055*	
C34	0.4752 (2)	0.2221 (2)	0.3806 (2)	0.0370 (5)	
H34A	0.4504	0.1585	0.4399	0.055*	
H34B	0.4065	0.2778	0.3260	0.055*	
H34C	0.4978	0.2676	0.4151	0.055*	

### Atomic displacement parameters (Å^2^)

**Table:**

	*U*^11^	*U*^22^	*U*^33^	*U*^12^	*U*^13^	*U*^23^
I1	0.02965 (8)	0.04227 (9)	0.03667 (8)	−0.01834 (6)	0.01302 (6)	−0.01549 (7)
I2	0.03260 (8)	0.04483 (9)	0.03496 (8)	−0.02140 (7)	0.00914 (6)	−0.01315 (7)
S1	0.0195 (2)	0.0205 (2)	0.0256 (2)	−0.00292 (17)	0.00131 (17)	−0.00429 (18)
S2	0.0218 (2)	0.0233 (2)	0.0259 (2)	−0.00656 (18)	0.00033 (18)	−0.00919 (18)
F1	0.0640 (12)	0.188 (3)	0.0504 (10)	−0.0825 (15)	0.0297 (9)	−0.0322 (13)
F2	0.0415 (8)	0.0622 (10)	0.0547 (9)	−0.0280 (7)	0.0253 (7)	−0.0214 (8)
O1	0.0223 (7)	0.0286 (7)	0.0228 (6)	−0.0104 (6)	−0.0003 (5)	−0.0042 (6)
O2	0.0287 (8)	0.0248 (7)	0.0361 (8)	−0.0061 (6)	0.0028 (6)	0.0004 (6)
O3	0.0226 (7)	0.0297 (8)	0.0369 (8)	−0.0030 (6)	0.0058 (6)	−0.0110 (6)
O4	0.0222 (7)	0.0291 (7)	0.0266 (7)	−0.0082 (6)	0.0005 (5)	−0.0074 (6)
O5	0.0281 (8)	0.0360 (8)	0.0377 (8)	−0.0025 (6)	0.0021 (6)	−0.0198 (7)
O6	0.0323 (8)	0.0290 (8)	0.0377 (8)	−0.0141 (6)	−0.0035 (6)	−0.0051 (6)
C1	0.0204 (9)	0.0228 (9)	0.0236 (9)	−0.0076 (7)	0.0031 (7)	−0.0076 (7)
C2	0.0194 (9)	0.0206 (9)	0.0257 (9)	−0.0057 (7)	0.0009 (7)	−0.0098 (7)
C3	0.0241 (9)	0.0218 (9)	0.0256 (9)	−0.0073 (7)	0.0025 (7)	−0.0074 (7)
C4	0.0235 (9)	0.0237 (9)	0.0311 (10)	−0.0103 (8)	0.0070 (8)	−0.0130 (8)
C5	0.0203 (9)	0.0251 (9)	0.0392 (11)	−0.0077 (8)	0.0014 (8)	−0.0133 (9)
C6	0.0228 (9)	0.0242 (9)	0.0308 (10)	−0.0066 (8)	−0.0038 (8)	−0.0057 (8)
C7	0.0234 (9)	0.0228 (9)	0.0235 (9)	−0.0094 (7)	0.0017 (7)	−0.0072 (7)
C8	0.0225 (9)	0.0260 (9)	0.0246 (9)	−0.0088 (8)	0.0002 (7)	−0.0098 (8)
C9	0.0251 (10)	0.0446 (12)	0.0204 (9)	−0.0172 (9)	0.0004 (7)	−0.0101 (9)
C10	0.0270 (11)	0.0594 (15)	0.0297 (11)	−0.0122 (11)	0.0021 (9)	−0.0196 (11)
C11	0.0269 (12)	0.107 (3)	0.0342 (13)	−0.0167 (14)	0.0046 (10)	−0.0342 (15)
C12	0.0439 (15)	0.118 (3)	0.0278 (12)	−0.0539 (18)	0.0096 (11)	−0.0165 (15)
C13	0.0576 (17)	0.084 (2)	0.0306 (12)	−0.0534 (17)	0.0019 (12)	−0.0057 (13)
C14	0.0442 (13)	0.0530 (14)	0.0263 (10)	−0.0310 (12)	−0.0024 (9)	−0.0061 (10)
C15	0.0256 (10)	0.0328 (11)	0.0277 (10)	−0.0045 (8)	−0.0064 (8)	−0.0077 (9)
C16	0.0403 (13)	0.0501 (14)	0.0318 (11)	−0.0132 (11)	0.0049 (10)	−0.0209 (11)
C17	0.0351 (12)	0.0357 (12)	0.0466 (13)	−0.0147 (10)	0.0017 (10)	−0.0168 (10)
C18	0.0217 (9)	0.0266 (9)	0.0241 (9)	−0.0081 (8)	0.0007 (7)	−0.0107 (8)
C19	0.0257 (10)	0.0256 (9)	0.0222 (9)	−0.0090 (8)	−0.0003 (7)	−0.0097 (8)
C20	0.0239 (10)	0.0301 (10)	0.0268 (9)	−0.0098 (8)	0.0012 (8)	−0.0116 (8)
C21	0.0291 (10)	0.0344 (11)	0.0280 (10)	−0.0155 (9)	0.0049 (8)	−0.0130 (9)
C22	0.0369 (12)	0.0332 (11)	0.0279 (10)	−0.0162 (9)	0.0020 (9)	−0.0060 (9)
C23	0.0299 (11)	0.0317 (11)	0.0303 (10)	−0.0092 (9)	−0.0027 (8)	−0.0072 (9)
C26	0.0209 (9)	0.0305 (10)	0.0252 (9)	−0.0088 (8)	0.0009 (7)	−0.0129 (8)
C24	0.0241 (10)	0.0310 (10)	0.0265 (9)	−0.0125 (8)	0.0030 (8)	−0.0110 (8)
C25	0.0261 (10)	0.0233 (9)	0.0244 (9)	−0.0075 (8)	−0.0011 (7)	−0.0103 (8)
C27	0.0233 (10)	0.0288 (10)	0.0364 (11)	−0.0084 (8)	0.0025 (8)	−0.0147 (9)
C28	0.0308 (11)	0.0320 (11)	0.0343 (11)	−0.0148 (9)	0.0009 (9)	−0.0084 (9)
C29	0.0275 (10)	0.0467 (13)	0.0303 (10)	−0.0199 (10)	0.0089 (8)	−0.0151 (10)
C30	0.0273 (11)	0.0399 (12)	0.0364 (11)	−0.0087 (9)	0.0072 (9)	−0.0201 (10)
C31	0.0268 (10)	0.0286 (10)	0.0339 (10)	−0.0090 (8)	0.0027 (8)	−0.0144 (9)
C32	0.0272 (10)	0.0282 (10)	0.0266 (9)	−0.0119 (8)	0.0005 (8)	−0.0110 (8)
C33	0.0315 (11)	0.0394 (12)	0.0364 (11)	−0.0100 (10)	−0.0066 (9)	−0.0129 (10)
C34	0.0345 (12)	0.0410 (12)	0.0374 (12)	−0.0066 (10)	0.0015 (9)	−0.0236 (10)

### Geometric parameters (Å, º)

**Table:**

I1—C4	2.0982 (19)	C15—C17	1.517 (3)
I1—O2^i^	3.3646 (15)	C15—C16	1.526 (3)
I2—C21	2.098 (2)	C15—H15	1.0000
I2—O3^ii^	3.2354 (14)	C16—H16A	0.9800
S1—O2	1.4399 (15)	C16—H16B	0.9800
S1—O3	1.4392 (14)	C16—H16C	0.9800
S1—C1	1.7450 (19)	C17—H17A	0.9800
S1—C15	1.787 (2)	C17—H17B	0.9800
S2—O6	1.4331 (16)	C17—H17C	0.9800
S2—O5	1.4400 (15)	C18—C25	1.371 (3)
S2—C18	1.751 (2)	C18—C19	1.448 (3)
S2—C32	1.791 (2)	C19—C24	1.389 (3)
F1—C12	1.357 (3)	C19—C20	1.395 (3)
F2—C29	1.354 (2)	C20—C21	1.383 (3)
O1—C8	1.367 (2)	C20—H20	0.9500
O1—C7	1.377 (2)	C21—C22	1.399 (3)
O4—C25	1.378 (2)	C22—C23	1.392 (3)
O4—C24	1.379 (2)	C22—H22	0.9500
C1—C8	1.361 (3)	C23—C24	1.377 (3)
C1—C2	1.454 (3)	C23—H23	0.9500
C2—C7	1.390 (3)	C26—C27	1.392 (3)
C2—C3	1.398 (3)	C26—C31	1.395 (3)
C3—C4	1.386 (3)	C26—C25	1.459 (3)
C3—H3	0.9500	C27—C28	1.385 (3)
C4—C5	1.396 (3)	C27—H27	0.9500
C5—C6	1.377 (3)	C28—C29	1.373 (3)
C5—H5	0.9500	C28—H28	0.9500
C6—C7	1.372 (3)	C29—C30	1.373 (3)
C6—H6	0.9500	C30—C31	1.385 (3)
C8—C9	1.467 (3)	C30—H30	0.9500
C9—C10	1.384 (3)	C31—H31	0.9500
C9—C14	1.402 (3)	C32—C34	1.522 (3)
C10—C11	1.400 (3)	C32—C33	1.526 (3)
C10—H10	0.9500	C32—H32	1.0000
C11—C12	1.361 (5)	C33—H33A	0.9800
C11—H11	0.9500	C33—H33B	0.9800
C12—C13	1.362 (5)	C33—H33C	0.9800
C13—C14	1.379 (3)	C34—H34A	0.9800
C13—H13	0.9500	C34—H34B	0.9800
C14—H14	0.9500	C34—H34C	0.9800
			
C4—I1—O2^i^	152.77 (6)	H16A—C16—H16C	109.5
C21—I2—O3^ii^	170.66 (6)	H16B—C16—H16C	109.5
O2—S1—O3	117.87 (9)	C15—C17—H17A	109.5
O2—S1—C1	106.58 (9)	C15—C17—H17B	109.5
O3—S1—C1	109.02 (9)	H17A—C17—H17B	109.5
O2—S1—C15	108.55 (10)	C15—C17—H17C	109.5
O3—S1—C15	107.76 (10)	H17A—C17—H17C	109.5
C1—S1—C15	106.54 (9)	H17B—C17—H17C	109.5
O6—S2—O5	118.36 (10)	C25—C18—C19	107.14 (17)
O6—S2—C18	110.60 (9)	C25—C18—S2	129.91 (15)
O5—S2—C18	106.18 (9)	C19—C18—S2	122.84 (14)
O6—S2—C32	108.55 (9)	C24—C19—C20	119.06 (18)
O5—S2—C32	108.78 (9)	C24—C19—C18	105.28 (17)
C18—S2—C32	103.33 (9)	C20—C19—C18	135.65 (18)
C8—O1—C7	107.10 (14)	C21—C20—C19	117.35 (19)
C25—O4—C24	107.14 (15)	C21—C20—H20	121.3
C8—C1—C2	107.13 (17)	C19—C20—H20	121.3
C8—C1—S1	126.98 (15)	C20—C21—C22	122.63 (19)
C2—C1—S1	125.85 (14)	C20—C21—I2	118.79 (15)
C7—C2—C3	119.05 (17)	C22—C21—I2	118.57 (15)
C7—C2—C1	104.66 (16)	C23—C22—C21	120.3 (2)
C3—C2—C1	136.28 (18)	C23—C22—H22	119.9
C4—C3—C2	116.79 (18)	C21—C22—H22	119.9
C4—C3—H3	121.6	C24—C23—C22	116.2 (2)
C2—C3—H3	121.6	C24—C23—H23	121.9
C3—C4—C5	122.73 (18)	C22—C23—H23	121.9
C3—C4—I1	119.58 (15)	C27—C26—C31	119.35 (18)
C5—C4—I1	117.65 (14)	C27—C26—C25	121.84 (17)
C6—C5—C4	120.62 (18)	C31—C26—C25	118.78 (18)
C6—C5—H5	119.7	C23—C24—O4	125.19 (18)
C4—C5—H5	119.7	C23—C24—C19	124.40 (19)
C7—C6—C5	116.33 (18)	O4—C24—C19	110.35 (17)
C7—C6—H6	121.8	C18—C25—O4	110.08 (17)
C5—C6—H6	121.8	C18—C25—C26	136.33 (18)
C6—C7—O1	125.00 (17)	O4—C25—C26	113.56 (16)
C6—C7—C2	124.48 (18)	C28—C27—C26	120.40 (19)
O1—C7—C2	110.49 (16)	C28—C27—H27	119.8
C1—C8—O1	110.61 (16)	C26—C27—H27	119.8
C1—C8—C9	135.85 (18)	C29—C28—C27	118.4 (2)
O1—C8—C9	113.54 (16)	C29—C28—H28	120.8
C10—C9—C14	120.1 (2)	C27—C28—H28	120.8
C10—C9—C8	121.0 (2)	F2—C29—C30	118.2 (2)
C14—C9—C8	118.8 (2)	F2—C29—C28	118.6 (2)
C9—C10—C11	119.1 (3)	C30—C29—C28	123.15 (19)
C9—C10—H10	120.5	C29—C30—C31	118.14 (19)
C11—C10—H10	120.5	C29—C30—H30	120.9
C12—C11—C10	118.9 (3)	C31—C30—H30	120.9
C12—C11—H11	120.6	C30—C31—C26	120.6 (2)
C10—C11—H11	120.6	C30—C31—H31	119.7
C11—C12—F1	117.2 (3)	C26—C31—H31	119.7
C11—C12—C13	123.5 (2)	C34—C32—C33	111.88 (17)
F1—C12—C13	119.3 (3)	C34—C32—S2	110.03 (15)
C12—C13—C14	118.3 (3)	C33—C32—S2	108.70 (14)
C12—C13—H13	120.8	C34—C32—H32	108.7
C14—C13—H13	120.8	C33—C32—H32	108.7
C13—C14—C9	120.2 (3)	S2—C32—H32	108.7
C13—C14—H14	119.9	C32—C33—H33A	109.5
C9—C14—H14	119.9	C32—C33—H33B	109.5
C17—C15—C16	113.35 (19)	H33A—C33—H33B	109.5
C17—C15—S1	110.96 (15)	C32—C33—H33C	109.5
C16—C15—S1	110.46 (16)	H33A—C33—H33C	109.5
C17—C15—H15	107.3	H33B—C33—H33C	109.5
C16—C15—H15	107.3	C32—C34—H34A	109.5
S1—C15—H15	107.3	C32—C34—H34B	109.5
C15—C16—H16A	109.5	H34A—C34—H34B	109.5
C15—C16—H16B	109.5	C32—C34—H34C	109.5
H16A—C16—H16B	109.5	H34A—C34—H34C	109.5
C15—C16—H16C	109.5	H34B—C34—H34C	109.5
			
O2—S1—C1—C8	−152.76 (18)	O6—S2—C18—C25	17.2 (2)
O3—S1—C1—C8	−24.6 (2)	O5—S2—C18—C25	146.80 (18)
C15—S1—C1—C8	91.48 (19)	C32—S2—C18—C25	−98.80 (19)
O2—S1—C1—C2	29.66 (19)	O6—S2—C18—C19	−167.17 (15)
O3—S1—C1—C2	157.87 (16)	O5—S2—C18—C19	−37.57 (18)
C15—S1—C1—C2	−86.10 (18)	C32—S2—C18—C19	76.83 (17)
C8—C1—C2—C7	−0.2 (2)	C25—C18—C19—C24	−1.1 (2)
S1—C1—C2—C7	177.74 (14)	S2—C18—C19—C24	−177.63 (14)
C8—C1—C2—C3	178.5 (2)	C25—C18—C19—C20	178.6 (2)
S1—C1—C2—C3	−3.6 (3)	S2—C18—C19—C20	2.1 (3)
C7—C2—C3—C4	1.0 (3)	C24—C19—C20—C21	−1.9 (3)
C1—C2—C3—C4	−177.6 (2)	C18—C19—C20—C21	178.4 (2)
C2—C3—C4—C5	−0.5 (3)	C19—C20—C21—C22	−0.5 (3)
C2—C3—C4—I1	177.18 (13)	C19—C20—C21—I2	−179.34 (14)
O2^i^—I1—C4—C3	−102.08 (18)	O3^ii^—I2—C21—C20	−30.8 (5)
O2^i^—I1—C4—C5	75.7 (2)	O3^ii^—I2—C21—C22	150.3 (3)
C3—C4—C5—C6	−0.4 (3)	C20—C21—C22—C23	1.5 (3)
I1—C4—C5—C6	−178.08 (15)	I2—C21—C22—C23	−179.71 (16)
C4—C5—C6—C7	0.7 (3)	C21—C22—C23—C24	0.1 (3)
C5—C6—C7—O1	177.68 (18)	C22—C23—C24—O4	−179.66 (19)
C5—C6—C7—C2	−0.1 (3)	C22—C23—C24—C19	−2.7 (3)
C8—O1—C7—C6	−178.10 (19)	C25—O4—C24—C23	177.04 (19)
C8—O1—C7—C2	0.0 (2)	C25—O4—C24—C19	−0.2 (2)
C3—C2—C7—C6	−0.7 (3)	C20—C19—C24—C23	3.7 (3)
C1—C2—C7—C6	178.25 (18)	C18—C19—C24—C23	−176.46 (19)
C3—C2—C7—O1	−178.81 (16)	C20—C19—C24—O4	−178.95 (16)
C1—C2—C7—O1	0.2 (2)	C18—C19—C24—O4	0.8 (2)
C2—C1—C8—O1	0.2 (2)	C19—C18—C25—O4	1.0 (2)
S1—C1—C8—O1	−177.71 (14)	S2—C18—C25—O4	177.20 (14)
C2—C1—C8—C9	−179.6 (2)	C19—C18—C25—C26	−176.7 (2)
S1—C1—C8—C9	2.4 (3)	S2—C18—C25—C26	−0.5 (3)
C7—O1—C8—C1	−0.1 (2)	C24—O4—C25—C18	−0.5 (2)
C7—O1—C8—C9	179.75 (16)	C24—O4—C25—C26	177.77 (15)
C1—C8—C9—C10	59.0 (3)	C27—C26—C25—C18	−46.5 (3)
O1—C8—C9—C10	−120.9 (2)	C31—C26—C25—C18	135.5 (2)
C1—C8—C9—C14	−124.5 (3)	C27—C26—C25—O4	135.87 (19)
O1—C8—C9—C14	55.7 (2)	C31—C26—C25—O4	−42.1 (2)
C14—C9—C10—C11	0.1 (3)	C31—C26—C27—C28	−0.2 (3)
C8—C9—C10—C11	176.64 (19)	C25—C26—C27—C28	−178.22 (19)
C9—C10—C11—C12	−0.2 (3)	C26—C27—C28—C29	−0.1 (3)
C10—C11—C12—F1	−179.8 (2)	C27—C28—C29—F2	−179.78 (19)
C10—C11—C12—C13	0.0 (4)	C27—C28—C29—C30	0.1 (3)
C11—C12—C13—C14	0.3 (4)	F2—C29—C30—C31	−179.8 (2)
F1—C12—C13—C14	−179.9 (2)	C28—C29—C30—C31	0.3 (3)
C12—C13—C14—C9	−0.4 (4)	C29—C30—C31—C26	−0.7 (3)
C10—C9—C14—C13	0.2 (3)	C27—C26—C31—C30	0.7 (3)
C8—C9—C14—C13	−176.4 (2)	C25—C26—C31—C30	178.72 (19)
O2—S1—C15—C17	−178.23 (14)	O6—S2—C32—C34	−52.80 (17)
O3—S1—C15—C17	53.08 (17)	O5—S2—C32—C34	177.16 (15)
C1—S1—C15—C17	−63.79 (17)	C18—S2—C32—C34	64.64 (16)
O2—S1—C15—C16	−51.66 (17)	O6—S2—C32—C33	70.04 (16)
O3—S1—C15—C16	179.65 (15)	O5—S2—C32—C33	−60.00 (17)
C1—S1—C15—C16	62.77 (17)	C18—S2—C32—C33	−172.52 (15)

Symmetry codes: (i) −*x*+1, −*y*+1, −*z*+1; (ii) −*x*+2, −*y*+1, −*z*.

### Hydrogen-bond geometry (Å, º)

**Table:**

*D*—H···*A*	*D*—H	H···*A*	*D*···*A*	*D*—H···*A*
C6—H6···O1^iii^	0.95	2.56	3.418 (2)	150
C15—H15···O2^iv^	1.00	2.52	3.520 (3)	177
C30—H30···O2^v^	0.95	2.58	3.463 (3)	155
C31—H31···O3^v^	0.95	2.58	3.260 (2)	129

Symmetry codes: (iii) −*x*+1, −*y*+2, −*z*; (iv) −*x*+2, −*y*+1, −*z*+1; (v) *x*−1, *y*, *z*.
